# Charge Transfer Dynamics at Dye-Sensitized ZnO and TiO_2_ Interfaces Studied by Ultrafast XUV Photoelectron Spectroscopy

**DOI:** 10.1038/srep24422

**Published:** 2016-04-13

**Authors:** Mario Borgwardt, Martin Wilke, Thorsten Kampen, Sven Mähl, Manda Xiao, Leone Spiccia, Kathrin M. Lange, Igor Yu. Kiyan, Emad F. Aziz

**Affiliations:** 1 Joint Laboratory for Ultrafast Dynamics in Solutions and at Interfaces (JULiq), Institute of Methods for Material Development, Helmholtz-Zentrum Berlin, Albert-Einstein-Strasse 15, D-12489 Berlin, Germany; 2 Department of Physics, Freie Universität Berlin, Arnimallee 14, 14195 Berlin, Germany; 3 SPECS Surface Nano Analysis GmbH, Voltastrasse 5, D-13355 Berlin, Germany; 4 School of Chemistry, Monash University, Clayton 3800, VIC, Australia; 5 Institute of Solar Fuels, Helmholtz-Zentrum Berlin, Albert-Einstein-Strasse 15, D-12489 Berlin, Germany

## Abstract

Interfacial charge transfer from photoexcited ruthenium-based N3 dye molecules into ZnO thin films received controversial interpretations. To identify the physical origin for the delayed electron transfer in ZnO compared to TiO_2_, we probe directly the electronic structure at both dye-semiconductor interfaces by applying ultrafast XUV photoemission spectroscopy. In the range of pump-probe time delays between 0.5 to 1.0 ps, the transient signal of the intermediate states was compared, revealing a distinct difference in their electron binding energies of 0.4 eV. This finding strongly indicates the nature of the charge injection at the ZnO interface associated with the formation of an interfacial electron-cation complex. It further highlights that the energetic alignment between the dye donor and semiconductor acceptor states appears to be of minor importance for the injection kinetics and that the injection efficiency is dominated by the electronic coupling.

Ultrafast photoinduced electron transfer is a fundamental process occurring in a wide range of nanostructured materials used in photovoltaic devices or for photocatalytic solar fuel generation^1^,^2^,^3^. A detailed understanding of this fundamental mechanism is important for the future development and design of efficient systems for sustainable solar energy conversion. In particular, the electron injection in dye-sensitized solar cells (DSSCs) from a photoexcited dye into a wide bandgap semiconductor, e.g. TiO_2_, ZnO or SnO_2_, has been of much interest in the last decades^4^,^5^,^6^,^7^,^8^. In such an electrochemical device, sunlight is converted into electricity due to efficient absorption of photons by a panchromatic dye and subsequent charge separation at the dye-semiconductor interface. Among numerous sensitizer-semiconductor combinations, ruthenium(II) complexes such as [Ru(dcbpyH)_2_(NCS)_2_] (commonly termed N3), where dcbpy is 4,4′-dicarboxy-2,2′-bipyridine, and N719, the corresponding bis(tetrabutyl)ammonium salt, were found to possess a high electron transfer probability (close to one) in the visible range of the solar spectrum when the dye is attached to a TiO_2_ substrate^9^,^10^,^11^. Therefore, TiO_2_-based DSSCs, exhibiting a solar-to-electric energy conversion efficiency above 10%, are considered as a reference in the study of fundamental aspects of photoinduced charge transfer.

Zinc oxide (ZnO) represents a promising alternative to TiO_2_ due to its much higher bulk electron mobility^12^,^13^ and great diversity in the nanostructured electrodes that can be produced^14^,^15^, e.g., nanoparticles^16^,^17^, nanorods^18^ and nanosheets^19^. However, the achieved energy conversion efficiencies of ZnO-based DSSCs are still much below the record values of their TiO_2_-based counterparts^20^. This issue initiated a long-lasting discussion on the possible reasons. Ultrafast transient absorption spectroscopy has revealed that the injection kinetics in ZnO-based substrates occurs on a different, picosecond, timescale^21^,^22^,^23^,^24^,^25^,^26^,^27^,^28^,^29^, whereas the charge separation at TiO_2_ interfaces is by order of magnitudes faster and takes place in a femtosecond time domain^30^,^31^,^32^,^33^,^34^,^35^,^36^,^37^. The slower dynamic response is, therefore, often related to the limited performance of ZnO-based cells, but its physical origin is still not fully understood. Both semiconductors possess similar band gap and conduction band positions so that the energy level alignment cannot explain these findings. However, the electronic properties of the conduction band determine the coupling between the dye and the semiconductor and, thus, represent a critical factor for the injection process.

For the charge transfer at the TiO_2_ interface, the well-established two-state injection model is used to describe the coexistence of a dominant ultrafast (<100 fs) injection and one or more slower minor components occurring on a picosecond timescale^38^. According to this model, the initially excited singlet metal-to-ligand charge-transfer-state (^1^MLCT) directly injects electrons as free mobile charge carriers to the conduction band of TiO_2_ on an ultrafast time scale^35^. This process occurs simultaneously with a rapid intramolecular relaxation into the triplet ^3^MLCT states via intersystem crossing on a similar timescale of 50–100 fs^34^,^35^. The subsequent injection from the relaxed triplet state is significantly slower and takes place on a picosecond timescale^39^. The slow injection from the ^3^MLCT state was argued to be caused by an unfavorable energetic band alignment, where the donor states largely lie below the acceptor states and the electron transfer is possible only due to a partial overlap of the ^3^MLCT band and the conduction band^40^.

Two competing descriptions have been proposed to account for the slow character of injection kinetics at the ZnO interface. One mechanism is based on an adapted two-state injection model^26^ where the direct ultrafast electron transfer from the ^1^MLCT is considered to be highly suppressed (see Fig. 1a). It follows that the majority of the injected electrons originates from the ^3^MLCT state. In this representation, the retained electrons reside on the dye before they become free mobile charge carriers. A second completely different injection mechanism, consisting of the formation of an interfacial electron-cation complex (IC), was suggested on the basis of a variety of experimental results (see Fig. 1b). It was shown that an increase in the yield of positive dye cations is not necessary directly related to the release of mobile electrons^22^,^24^. Thus, the electrons are considered to be temporarily delayed at the dye-ZnO interface via the IC formation. The second description does not exclude an ultrafast depopulation of the ^1^MLCT state via electron ejection from the dye but rather suggests a mechanism of the carrier-formation delay. The possible origin of the IC has been addressed before. Furube *et al.*^22^ proposed that hybridization of the molecular excited states with the surface-localized ZnO states leads to the formation of a new state, called “exciplex”, and further explained that this could be an electron-donor (D) and acceptor (A) pair occurring in a neutral (D * A) or ionic (D + A−) state. This consideration was further corroborated in refs 24,27, where the strong electrostatic interaction between the cations and injected electrons at the surface was argued to stabilize the intermediate state. Due to the lower electrostatic screening in ZnO as compared to TiO_2_, such stabilization is more efficient in the former case, which might explain that the IC can be found only at the ZnO interface. For both competing descriptions shown in Fig. 1, the intermediate state is populated on a timescale below 500 fs and has a lifetime in the picosecond domain.

So far, the involved transient states were probed indirectly by means of time-resolved absorption spectroscopy applied in different ranges of photon energy. However, the transient absorption signal in the visible and IR domain is rather difficult to interpret due to the co-existence of broad and overlapping absorption bands^22^,^26^. Time-resolved inner-shell photoelectron spectroscopy seems to be a promising approach to identify the suitable injection mechanism^41^,^42^. A drawback of this approach is that the interpretation of the transient signals relies on the support from complex time-dependent theoretical modeling.

In the present work, time-resolved photoelectron spectroscopy (PES) in the XUV energy range is applied to probe directly the population dynamics of the intermediate states and their binding energies^43^. By comparing results obtained for TiO_2_ and ZnO substrates, the unique properties of the latter interface are revealed. In a previous study^40^, we have already demonstrated the capabilities of this method in both revealing the injection kinetics and determining the binding energies of the involved states. In the present experiment, N3 molecules are excited by an optical pump pulse, close to the absorption maximum of the dye (*λ* ~ 530 nm), and the evolution of the excited system is followed by monitoring the transient changes in the electron kinetic energy spectra.

## Results and Discussion

**Figure 2:**
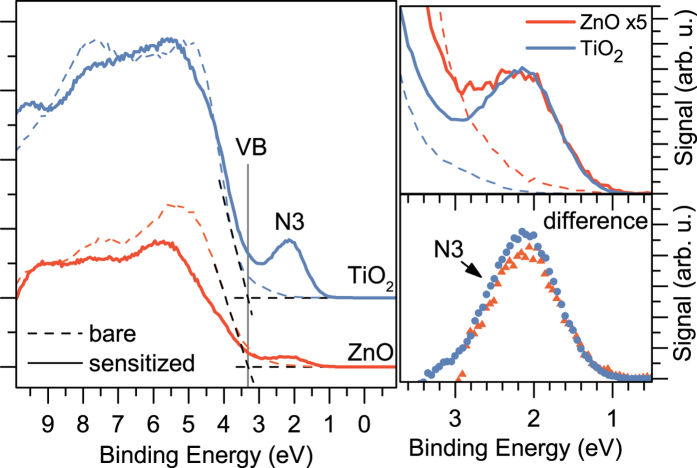
(Left) Steady-state XUV spectra of photoelectrons recorded for the bare (dashed lines) and sensitized (solid lines) ZnO and TiO_2_ samples. The emission peak from the dye ground state and the valence band maxiumum are labeled by N3 and VB, respectively. (Right) The upper panel shows in detail the ionization contribution from the N3 HOMO band on both substrates. The signal for the bare and sensitized ZnO electrode was multiplied by a factor of five. The lower panel shows the difference in the emission yield between the sensitized and bare substrates for ZnO and TiO_2_, respectively.

Figure 2 shows the XUV photoelectron spectra in the range of binding energies of the semiconductor valence band and the N3-dye HOMO band, recorded for the bare and sensitized TiO_2_ and ZnO substrates, respectively, without application of the pump beam and with equal acquisition times. The energy positions of the higher-lying electronic structure components, which are important for the photoinduced electron transfer at the dye-semiconductor interfaces, can be estimated from these measurements. The valence-band maxima of the non-sensitized substrates can be seen to lie at approximately the same binding energy of 3.3 eV. By taking the optical band gap of 3.3 eV into account, we find that for both substrates the conduction band minimum lies close to the Fermi level^44^. The right panel of Fig. 2 shows the spectra in the binding energy range encompassing the N3-dye HOMO band. The band shape appears to be very similar for both substrates and its maximum arises at a binding energy of 2.1 eV. This value is in agreement with the previously reported results for the N3-sensitized interfaces^45^,^46^,^47^. In order to obtain an equal ionization yield of the dye on both substrates, the signal from ZnO/N3 interface was multiplied by a factor of five. Since the ionization cross section of N3 is expected to be the same, this implies that TiO_2_ and ZnO substrates exhibit different surface coverage, which can be easily explained by a difference in the surface morphology and sensitization time.

**Figure 3:**
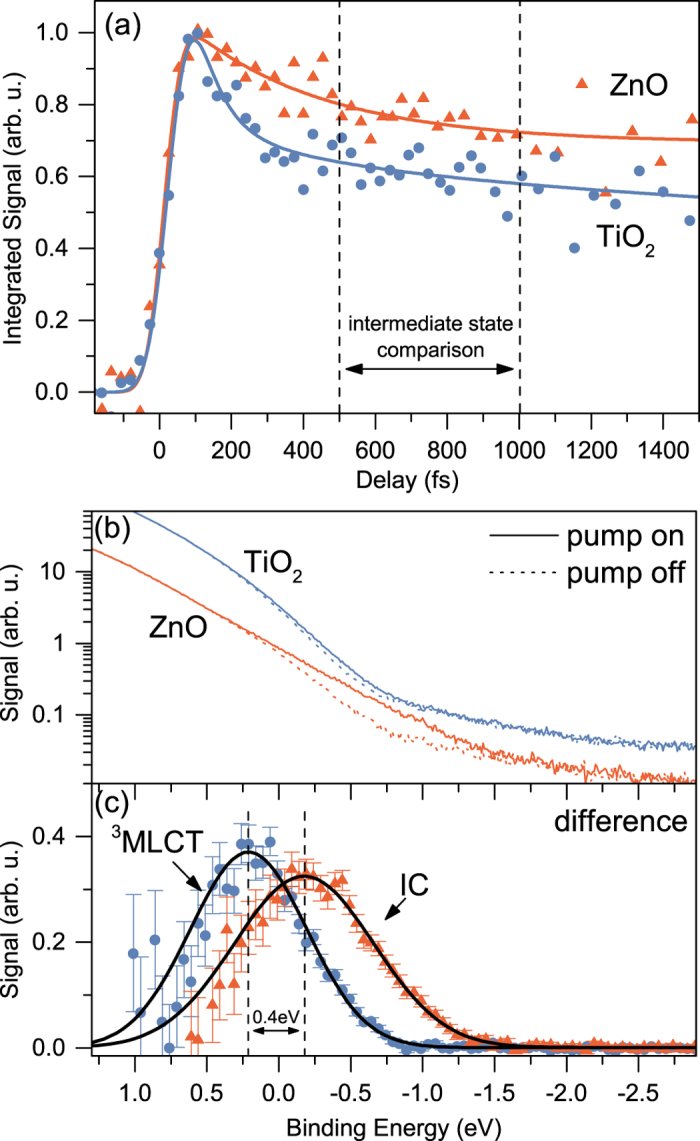
Transient signal of the sensitized ZnO and TiO_2_ substrates. (**a**) Dependence of integrated electron yield of the background-subtracted XUV spectra on the pump-probe time delay. The solid lines represent results of fit to a sum of exponential functions convoluted with the system response function (see text). (**b**) Transient spectra averaged over pump-probe time delays between 0.5 to 1.0 ps (pump on) compared to the background steady-state spectra (pump off). (**c**) Difference of pump-on and pump-off spectra for both samples.

Figure 3a shows the transient photoemission signal recorded for both sensitized interfaces. The signal represents the electron yield integrated over the range of binding energies between −2 and 1 eV in the background-subtracted XUV spectra. This range lies above the HOMO band of N3 and encompasses the ionization contributions from transient states excited by the pump beam. Pump-probe spectra recorded at negative time delays were used as a background in the subtraction routine. Both transients shown in Fig. 3a were obtained by applying the same pump intensity and their maxima were normalized to unity.

A comparison of the two dependencies shown in Fig. 3a reveals a more complex population dynamics of transient states at the TiO_2_/N3 interface, manifesting a diverse injection mechanism as compared to ZnO/N3. Although the decay is multi-exponential in both cases, the initial fast component is not as pronounced for ZnO/N3. The different ratio between the fast and slow decay components for the two samples was already reported in literature^27^. In order to characterize the decay dynamics, the time dependence of the transient signal was fitted to a sum of exponential functions, convoluted with a Gaussian profile of 105 fs width representing the system response. A sum of two and three exponential functions was needed to reproduce results obtained for ZnO/N3 and TiO_2_/N3 interfaces, respectively. In case of TiO_2_, the fastest decay process is characterized by a lifetime constant of 35 ± 20 fs. This process is not apparent in the ZnO transient. Notably, after this ultra-fast process, a similar time dependence of the transient states population is observed for both systems, i.e., two exponential decay processes; one with a half-life of hundreds of fs and the other slower process with a lifetime >50 ps. This finding is in agreement with the literature values which concluded that the formation of the intermediate state takes place within 500 fs after interaction with a short pump pulse^22^. Therefore, our further analysis of the binding energies of transient states is carried out for pump-probe time delays in the range between 0.5 and 1 ps. To gain a better signal-to-noise ratio, the transient XUV spectra were averaged over this range of delays.

Figure 3b shows the delay-averaged XUV spectra for both substrates as well as the corresponding background spectra obtained without applying the pump beam. The background-subtracted kinetic energy distributions are presented in Fig. 3c. One can see that in the given range of time delays ionization from a single band constitutes the transient photoemission yield for both interfaces. However, the binding energies of the transient states are rather different and lie at 0.2 ± 0.1 and −0.2 ± 0.1 eV for TiO_2_/N3 and ZnO/N3, respectively. The large energy shift of 0.4 eV indicates that the origin of the states is different. If the intermediate state of ZnO/N3 would be the ^3^MLCT state, as in the case of the TiO_2_/N3 interface, one should expect equal binding energies of the transient states in the 0.5–1 ps range of time delays, as it was found in our previous work on the injection kinetics of TiO_2_ and SnO_2_ electrodes sensitized with the closely related N719 dye (doubly deprotonated version of N3)^40^. It was demonstrated that the longer lived transient states of both TiO_2_ and SnO_2_ substrates possess very similar binding energies and spectral bandwidths. Supported by numerical modeling of the transient signal, it was concluded that the slow injection pathway resulted from the ^3^MLCT state. The binding energy of 0.1 eV of the ^3^MLCT state, reported in ref. 40 for the TiO_2_/N719 interface, is reproduced within the error estimate in the present measurement conducted on the TiO_2_/N3 interface. However, the injection kinetics on ZnO/N3 can not be described by the two-state model used for TiO_2_/N3. Thus, one can conclude that the electron dynamics at the ZnO/N3 interface involves the formation of an interfacial electron-cation complex. The formation of this different state is further supported by the fact that the intermediate state of ZnO/N3 exhibits a larger spectral bandwidth of 1.1 eV, as compared to a bandwidth of 0.8 eV for the TiO_2_/N3 transient. These values were obtained by deconvolution of the apparatus response function from the energy dependences shown in Fig. 3c. A possible reason of the difference in bandwidth could be a more delocalized character of the interfacial complex as compared to the molecular electronic state.

Our results provide direct evidence that for ZnO a different charge transfer mechanism is involved in the electron injection process, as compared to TiO_2_ and SnO_2_. This issue was raised previously by Stockwell *et al.*^27^. Furthermore, our results reveal that the dye-semiconductor electronic coupling can be particularly important and that must be taken into consideration in addition to the energy level alignment when rationalizing the electronic properties of dye-semiconductor interfaces. Indeed, the energetic position of the long-lived transient state of ZnO/N3 lies well above the conduction band minimum. Despite this alignment being more favorable for fast electron injection, the experimentally measured rate of injection was much slower. This finding rules out the previous argumentation in terms of the low density of acceptor states, used to explain the slow injection from the relaxed excited donor states near the conduction band edge of TiO_2_^21^.

Since the intersystem crossing of the N3 dye has been found to occur on a time scale of 100 fs^34^,^35^ and we do not observe a signature of the ^3^MLCT state formation, we conclude that the IC formation has to be considerably faster than 100 fs. A similar conclusion was drawn on the basis of transient absorption measurements by Furube *et al.*^22^ as well as with the use of time-resolved inner-shell photoelectron spectroscopy by Siefermann *et al.*^42^. However, a much higher time resolution is required to reveal the IC formation dynamics in greater detail.

As mentioned before, the long-lived character of the intermediate state was considered to be the reason for the poor performance of the ZnO-based DSSCs. However, it was shown previously that the injection rate at the TiO_2_ surface can also dramatically decrease to similar values in the presence of an electrolyte, while still yielding high device efficiency^37^,^48^. Therefore, the lifetime of the intermediate state as well as the rate at which the dye returns to the ground state (recombination) are both crucial factors determining the electron transfer probability to the semiconductor. Presumably, the different electronic coupling of the ZnO/N3 interfacial state with the ground N3 molecular state leads to an increased recombination rate, resulting in a smaller electron-transfer efficiency. This is in accordance with the conclusion drawn by Sundström and co-workers^24^ who found likewise the cause of a lowered device efficiency of ZnO-based solar cells in an increased recombination rate.

## Summary

In conclusion, the present study provides direct evidence of the formation of interfacial electronic states at the dye-sensitized ZnO interface. This feature of ZnO, in comparison to other materials such as TiO_2_ and SnO_2_, gives rise to a specific electronic coupling which governs the charge transfer process at ZnO electrodes. The developed method of transient XUV photoelectron spectroscopy is demonstrated to be a powerful tool for exploring both the ultrafast electron dynamics at interfaces and the absolute binding energies of the involved states. The latter capability makes it advantageous in comparison with other developed techniques such as transient absorption spectroscopy in the near-infrared or terahertz regime. Due to the high surface sensitivity, the PES provides a valuable insight into the charge transfer dynamics at dye-semiconductor interfaces.

## Experimental Methods

### Sample Preparation

The ZnO and TiO_2_ films were prepared on FTO glass (TEC7 glass plates, Dyesol). An area of 4 × 4 mm^2^ size on the FTO layer was coated with the mesoporous films utilizing a commercial semiautomatic screen printer and a commercial printing pastes (TiO_2_ particles of 18 nm average size, PST-18 NR, JGC Catalysts and Chemicals) (ZnO particles of 100 nm average size, nanopowder, Sigma-Aldrich). Other chemicals were obtained from commercial suppliers as used as received. The N3 dye was purchased from Solaronix (Switzerland). The semi-conductor sensitization was carried out using a dye solution with 0.3 mM N3 dissolved in ethanol (Sigma-Aldrich, HPLC grade). Prior to sensitization, the films were sintered at 500 °C for 30 min, cooled to 80 °C and then immersed into the dye solution. The TiO_2_ electrodes were sensitized over night at room temperature. In case of ZnO, a reduced sensitization time of 30 min was chosen in order minimize the impact of the dye solution to the samples^49^. The films were then thoroughly rinsed with ethanol to remove unbound dye and were transferred in air into the experimental vacuum chamber. A typical residual gas pressure in the chamber was in the range of 10^−7^ mbar during the experiment. The samples were prepared immediately prior to measurements. Because of the *ex-situ* sample preparation, special attention was paid to possible surface contamination. To exclude any influence by contaminants, measurements on identically prepared un-sensitized samples were conducted (see Supplementary Information S1: Pump-probe measurements on bare substrates). Slight changes of the samples spectra were observed when exposed to the XUV beam on a time scale of several ours. Therefore the sample position was changed according to this finding. Detailed analysis of XUV induced sample damage can be found in Supplementary Information S2 (S2: Sample damage measurements and control).

### Time-Resolved PES Experiment

A Ti:sapphire laser system delivering 2.5 mJ, 800 nm, 25 fs pulses at a repetition rate of 5 kHz was used to generate the visible pump and the XUV probe beams. Approximately 60% of the laser output was split to generate XUV light via up-conversion of the fundamental laser frequency in the process of high-order harmonic generation (HHG)^50^,^51^. A detailed description and characterization of our HHG setup can be found elsewhere^43^. The 21st harmonic of the fundamental frequency, with a photon energy of 32.6 eV, was selected using a zone-plate monochromator. Another part of the laser output was used for pumping of an optical parametric amplifier (OPA) to generate pulses at 530 nm wavelength. The spot sizes of the pump and the probe beams at the sample were 500 *μ*m and 100 *μ*m, respectively. The pump pulse energy was attenuated to 1 *μ*J, corresponding to a photon flux of 1.3 × 10^15^ photons/cm^2^ in the interaction region. At this intensity no spectral shift due to the space charge effect was visible (further details are provided in Supplementary Information S3: Space charge effect). A computer-controlled delay stage was used in the pump beam path to vary the time delay between the pump and probe pulses. A time resolution of approximately 105 fs (FWHM) in the present experiment was inferred from a cross-correlation measurement carried out on the bTiO_2_ sample. The kinetic energy spectra of photoelectrons were measured with the use of a time-of-flight (TOF) electron spectrometer THEMIS 600 delivered by SPECS. The calibration of the spectrometer’s work function was carried out by measuring the Fermi edge of a gold sample. The binding energies reported in this work were obtained from the measured electron kinetic energies with the work function taken into account. The spectral energy resolution was mainly determined by the HHG bandwidth convoluted with the spectrometer resolution. An energy resolution of 0.1 eV was derived from a calibration measurement by recording a XUV ionization spectrum of argon gas.

## Additional Information

**How to cite this article**: Borgwardt, M. *et al.* Charge Transfer Dynamics at Dye-Sensitized ZnO and TiO_2_ Interfaces Studied by Ultrafast XUV Photoelectron Spectroscopy. *Sci. Rep.* **6**, 24422; doi: 10.1038/srep24422 (2016).

## Supplementary Material

Supplementary Information

## Figures and Tables

**Figure 1:**
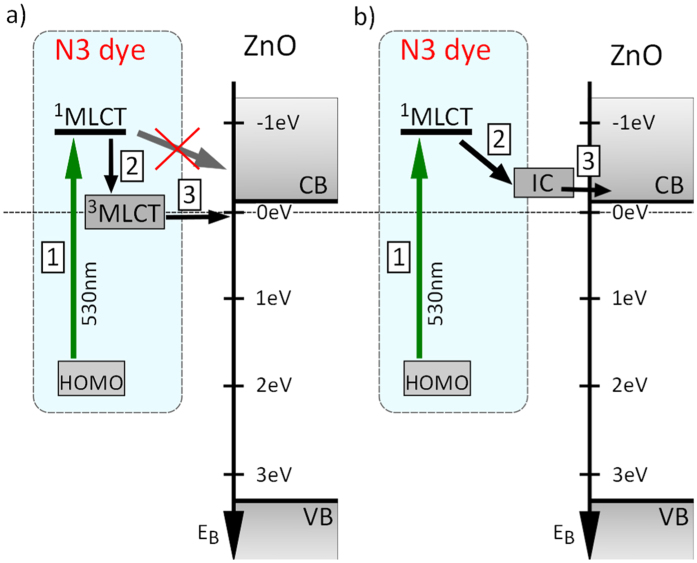
Schematic representation of the two models describing the charge transfer to ZnO after initial photoexcitation (1) of the N3 dye to the ^1^MLCT state. In (**a**) after internal relaxation (2) to the ^3^MLCT state charge transfer (3) occurs on a ps timescale. The electron is retained in the dye molecule and the ultrafast direct injection from ^1^MLCT, as compared to TiO_2_, is suppressed. In (**b**) the transfer process is mediated by the formation of the interfacial complex (2) followed by slow (ps timescale) charge transfer (3). In this model, the electron is retained at the interface between the dye and the semiconductor.
