# Supplementary material for: Charge Transfer Dynamics at Dye-Sensitized ZnO and TiO2 Interfaces Studied by Ultrafast XUV Photoelectron Spectroscopy
Source: Sci Rep. 2016 Apr 13;6:24422. doi: 10.1038/srep24422 (PMC4829909; doi:10.1038/srep24422)
Supplement: Supplementary Information [file srep24422-s1.pdf]

Supplementary Information:

Charge Transfer Dynamics at Dye-sensitized ZnO and TiO_2 Interfaces Studied by Ultrafast XUV Photoelectron Spectroscopy

M. Borgwardt,[†] Martin Wilke,[†] Thorsten Kampen,[¶] Sven Mähl,[¶] Manda Xiao,[§] Leone Spiccia,[§] Kathrin M. Lange,[‖] Igor Yu. Kiyan,[†] and Emad F. Aziz[*,†]

Joint Laboratory for Ultrafast Dynamics in Solutions and at Interfaces (JULiq), Institute of Methods for Material Development, Helmholtz-Zentrum Berlin, Albert-Einstein-Strasse 15, D-12489 Berlin, Germany, Department of Physics, Freie Universität Berlin, Arnimallee 14, 14195 Berlin,Germany, SPECS Surface Nano Analysis GmbH, Voltastrasse 5, D-13355 Berlin, Germany, School of Chemistry, Monash University, Clayton 3800, VIC, Australia, and Institute of Solar Fuels, Helmholtz-Zentrum Berlin, Albert-Einstein-Strasse 15, D-12489 Berlin, Germany

E-mail: emad.aziz@helmholtz-berlin.de

[*]To whom correspondence should be addressed

[†]Joint Laboratory for Ultrafast Dynamics in Solutions and at Interfaces (JULiq), Institute of Methods for Material Development, Helmholtz-Zentrum Berlin, Albert-Einstein-Strasse 15, D-12489 Berlin, Germany

[‡]Department of Physics, Freie Universität Berlin, Arnimallee 14, 14195 Berlin,Germany

[¶]SPECS Surface Nano Analysis GmbH, Voltastrasse 5, D-13355 Berlin, Germany

[§]School of Chemistry, Monash University, Clayton 3800, VIC, Australia

[‖]Institute of Solar Fuels, Helmholtz-Zentrum Berlin, Albert-Einstein-Strasse 15, D-12489 Berlin, Germany

S1: Pump-probe measurements on bare substrates

To reveal possible contributions from the substrates to the transient signal of the sensitized samples, pump-probe measurements on the bare substrates ($bTiO_2$ and bZnO) were conducted. These test measurements were used to determine the experimental time resolution as well as to ensure that the presence of contamination is negligible. Without the presence of resonant excited states, the process of non-resonant multiphoton ionization occurs when pump and probe pulses overlap in time. This effect, also called laser-assisted photoelectric effect from a surface[1,2] involves absorption of an XUV probe photon simultaneously with absorption or emission of one or more pump photons. Hence, sidebands in the XUV photoemission spectrum arise and their magnitude as a function of the pump-probe time delay represents a cross correlation (CC) signal of the pump and the probe beams. The CC width, determined by the widths convolution of the pump and probe pulses, constitutes the time resolution. The CC signal recorded with the bZnO sample is shown in Fig. S1. A fit of the experimental data to a Gaussian profile yields a width of 105 fs (FWHM). Similar results were obtained for the $bTiO_2$ substrate.

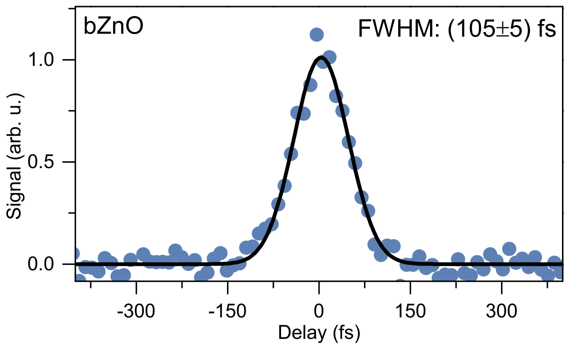

Figure S1: Pump-probe measurement on the bZnO sample. The transient background-subtracted signal is fitted to a Gaussian profile of 105 fs width (FWHM).

S2: Sample damage measurements and control

The sample damage may occur when a light-sensitive solid target is illuminated by a short laser pulse of high peak intensity. This issue is especially important when XUV radiation is applied, giving rise to a high ionization efficiency. Therefore, a sample-damage analysis was carried out in the present experiment. Figs. S2 (a) and (b) demonstrate the spectral change for the bare bZnO and the sensitized N3/ZnO samples, respectively, after their exposure to the XUV beam for 10 hours. In both cases changes in the XUV spectra are observed. To quantify the damage effect as a function of time and to disentangle the individual changes of the substrate and the dye, the photoemission yield in both bZnO and N3/ZnO spectra was integrated over the range of binding energies between 4.5 and 0 eV (see Fig S2 (c)). This range encompasses the ionization contributions from the HOMO band of N3 and is relevant to assess any degradation of the dye. One can see from Fig S2 (c) that the integrated yield obtained for bZnO is monotonically increasing, whereas for the sensitized sample the dependency appears to be more complex due to a superposition of yields arising from the bare substrate and the dye. Assuming a linear superposition of the yields, the difference between the two dependences demonstrates the change in the HOMO band signal related to a degradation of the dye (see green line in Fig S2 (c)). On the basis of this result we limited the maximum exposure time of an individual sample spot to 4 hours, corresponding to 10 % decrease of the dyes signal. A similar degradation study was carried out when the pump beam was applied. In this case no additional spectral changes were observed, which implies that sample degradation was caused mainly by the XUV probe beam.

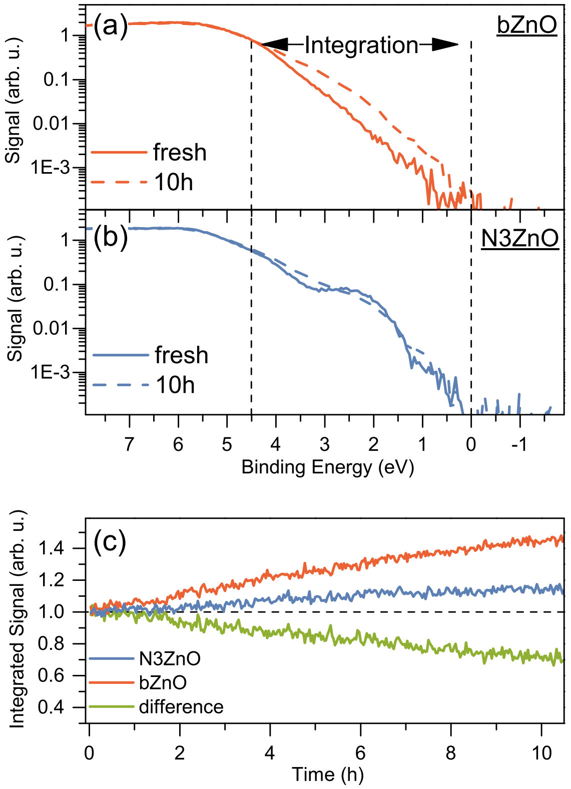

Figure S2: Steady-state XUV spectra of the bare ZnO (a) and sensitized N3/ZnO (b) samples, obtained in the beginning of exposure to the XUV beam (solid line) and after an exposure time of 10 hours (dashed line). Panel (c): ionization yield integrated over the range of binding energies between 4.5eV to 0 eV as a function of the XUV exposure time. The red and blue lines show the yields for the bare and the sensitized samples, respectively, and the green line shows the yield difference.

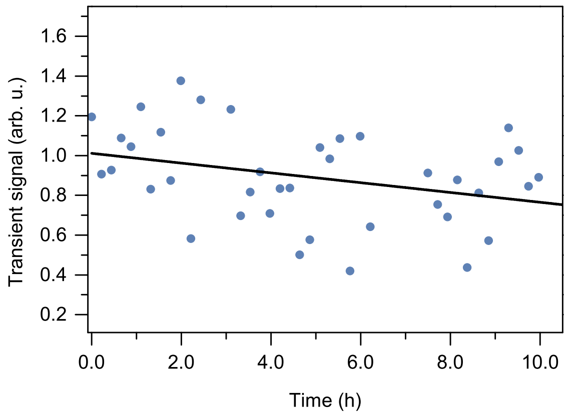

Figure S3: Normalized amplitude of the transient signal for N3/ZnO as a function of the XUV exposure time.

In addition, the amplitude of the transient signal for N3/ZnO was recorded during the

same time span of 10 hours. The normalized amplitude as a function of the exposure time is shown in Fig.S3. One can see that the decrease in the transient signal is comparable to the decrease in the steady-state photoemission yield of N3 shown in Fig S2 (c). However, the injection kinetics was found to be unaffected by the sample degradation.

S3: Space Charge Effect

The space-charge effect (SCE) arising from the interaction between pump and probe photoelectron clouds leads to a distortion of kinetic energy spectra and, thus, represents a limiting factor in time-resolved photoelectron spectroscopy.[3–5] In the present study, the pump beam was attenuated so that the spectral distortion was negligible. Figure S4 demonstrates the appearance of the SCE effect in XUV spectra obtained for three different values of the pump pulse energy. For the pulse energy of 1 μJ, corresponding to a peak intensity of 10^{10} W/cm^2 of the pump beam, the SCE effect can be neglected. This pump intensity was, therefore, applied in all pump-probe measurements.

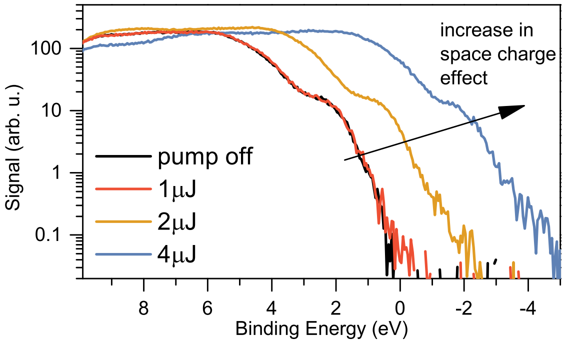

Figure S4: Transient XUV spectra of the sensitized ZnO sample recorded for three different values of the pump pulse energy: 1 μJ (red), 2 μJ (orange), and 4 μJ (blue). The black line shows a steady-state XUV spectrum.

References

(1) Miaja-Avila, L.; Lei, C.; Aeschlimann, M.; Gland, J. L.; Murnane, M. M.; Kapteyn, H. C.; Saathoff, G. Laser-Assisted Photoelectric Effect from Surfaces. *Physical*

Review Letters **2006**, *97*, 113604.

(2) Saathoff, G.; Miaja-Avila, L.; Aeschlimann, M.; Murnane, M. M.; Kapteyn, H. C. Laser-assisted photoemission from surfaces. *Physical Review A* **2008**, *77*, 022903.

(3) Al-Obaidi, R.; Wilke, M.; Borgwardt, M.; Metje, J.; Moguilevski, A.; Engel, N.; Tolksdorf, D.; Raheem, A.; Kampen, T.; Mhl, S. et al. Ultrafast photoelectron spectroscopy of solutions: space-charge effect. *New Journal of Physics* **2015**, *17*, 093016.

(4) Siffalovic, P.; Drescher, M.; Heinzmann, U. Femtosecond time-resolved core-level photoelectron spectroscopy tracking surface photovoltage transients on pGaAs. *EPL (Europhysics Letters)* **2002**, *60*, 924.

(5) Frietsch, B.; Carley, R.; Dbrich, K.; Gahl, C.; Teichmann, M.; Schwarzkopf, O.; Wernet, P.; Weinelt, M. A high-order harmonic generation apparatus for time- and angle-resolved photoelectron spectroscopy. *Review of Scientific Instruments* **2013**, *84*, 075106.
